# COVID-19 incidence of poverty: How has disease affected the cost of purchasing food in Pakistan

**DOI:** 10.1016/j.pmedr.2023.102477

**Published:** 2023-10-14

**Authors:** Muhammad Aamir Shahzad, Lianfen Wang, Shengze Qin, Sha Zhou

**Affiliations:** a School of Economics and Trade, Hunan University, Shi Jiachong, Yuelu District, Changsha City, Hunan Province 410079, China; b School of Tourism Management, Wuhan Business University, Wuhan Economic and Technological Development Zone, 430056 Wuhan, China

**Keywords:** COVID-19 pandemic, Income effect, Food cost, Healthy diets, Dietary diversity, Poverty

## Abstract

COVID-19 harms health and income generation activities. The pandemic caused poverty, and food crisis in the most vulnerable and underprivileged segments. Economic turbulence and massive poverty during epidemic period probably resulted in short-term food insecurity and low food consumption. Based on these concerns, the current study examined how COVID-19 has impacted Pakistan's cost of purchasing food. The study surveyed total 1067 Punjab and Sindh residents in Pakistan, from July to October 2021. A structural equation model was used to examine the interrelationship among food intake, food purchasing cost, and health effects. We investigated whether people experienced substantial effects from the loss of a source of income, work hours, debt burden, and food inflation on their food intake. These circumstances all negatively affected food intake, reducing food consumption. Besides COVID-19′s effects on direct income, 41% more people fall into poverty and 23% can't afford healthy food. People's socioeconomic circumstances affects poverty levels and affordable healthy food costs. The cost of purchasing food is significantly correlated with health outcomes. A significant and positive correlation between COVID-19 and income effects, and a negative correlation between food consumption and adverse income effects was explored. In addition, people increased their demand for food assistance during COVID-19 to mitigate negative income shocks. People who cannot afford minimal food costs should be offered food through mobile vehicles or delivery channels in the short term. Cash transfers or subsidies could also be provided to the needy during crisis time.

## Highlights

• COVID-19 caused poverty, and food crisis in underprivileged segments.
• Loss of source of income, work hours, debt burden, and food inflation negatively affected food consumption.
• 41% more people fall into poverty, 23% can't afford healthy food and socioeconomic circumstances affects poverty levels.
• Positive correlation between COVID-19 and income, while negative between food consumption and adverse income effects.
• People increased their demand for food assistance during COVID-19 to mitigate negative income shocks.

## 1 Introduction

The Sustainable Development Goals (SDGs), which aim to end poverty and hunger by 2030, have been harmed by COVID-19, especially in developing nations (Kassy et al., 2021). COVID-19 containment policies caused double-digit inflation, food shortages, and income losses for disadvantaged families (Baker et al., 2020; Daher, 2001). The working class was especially affected by broad economic pressure (Vazirani and Bhattacharjee, 2022). The illness's indirect and direct impacts, preventive measures, and government spread control measures all contribute to COVID-19′s long-term economic and societal effects (Kilby and McWhirter, 2022). Many households struggle to acquire economical and nutritious food because of COVID-19. Food insecurity promotes behavioral and household stress and short- and long-term health risks. Money and food issues may coexist with job loss, having limited resources, and social isolation (Goyal et al., 2020). Malnutrition, food access and poverty are closely related issues (Ettling et al., 1994). Historically, infectious diseases like swine flu, malaria, Ebola, and others hit emerging and developed nations like the US, South Korea, China, and Canada severely (Brown et al., 2021, Brown, 2021, Joo et al., 2019, Neumann et al., 2005, Queiroz et al., 2022, Shen et al., 2021, Zhang et al., 2020). Infectious disease and perpetual poverty trap have significant associations for centuries (Bloom et al., 2003, Jong-wook, 2003). Research documented have investigated the COVID-19 effects on economies (Almeida et al., 2021, Barletta et al., 2022, Bonds et al., 2010, Chakraborty et al., 2010, Goenka and Liu, 2020, Kolahchi et al., 2021, Millett et al., 2020, Varona and Gonzales, 2021, Wei et al., 2021). However, previous research has not evaluated how COVID-19 affects food purchasing ability, consumption, and poverty. Thus, this study answers the research question: how has COVID-19 affected Pakistani food purchasing power? This research has three main goals: (1) quantify the effects of COVID-19 on poverty incidence; (2) calculate how many people cannot afford the minimum amount needed for a healthy diet based on current food expenditures; and (3) determine how food purchasing cost, consumption, and COVID-19 are related.

**Fig. 1:**
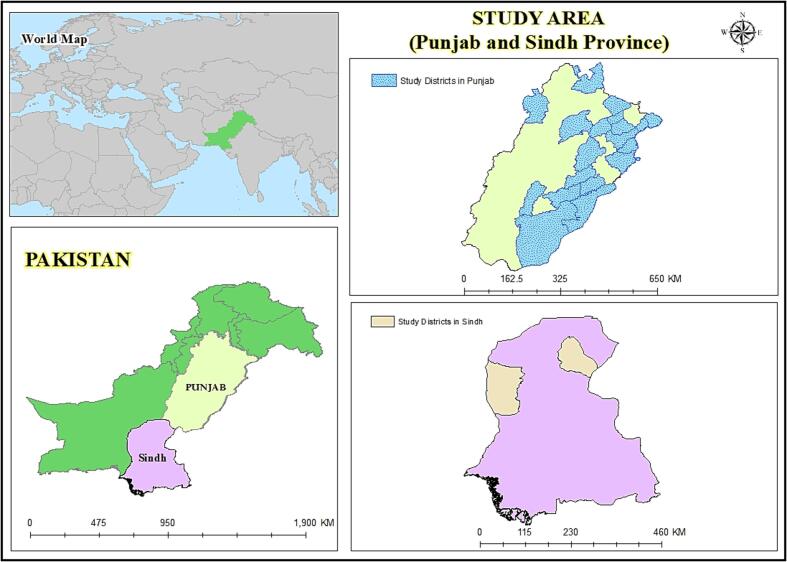
Map of the study area. *Note: The author takes the neutral stance is relation to territorial disputes or jurisdictional claims in its research.

This study adds to the body (Fig. 1) of knowledge on how the COVID-19 pandemic has affected grassroots food purchasing cost in Pakistan. Online surveys from the COVID-19 natural experiment were used to evaluate how the epidemic influences food cost and related factors. This research has broad public consequences. Economic growth alone cannot alleviate food security challenges. Income growth, nutrition policy measures, and health and investment (Table 1) policies are needed in combination (Béné, 2020, Grainger, 2010). In order to improve food security and purchasing power after the COVID-19 pandemic, policymakers and government agencies must develop and implement food security policies and practices.

**Table 1:** Distribution of district-wise study participants aged 17 years and older who participated in this study's online survey from Punjab and Sindh provinces in Pakistan during the COVID-19 pandemic period: July-October 2021.

Location	Frequency (N)	% age
Punjab province		
Vehari	200	18.74
Bahawalpur	200	18.74
Multan	200	18.74
Muzaffargarh	60	5.63
Sindh province		
Daddu	203	19.03
Sakhar	204	19.12
Total	1067	100

## 2 Material and methods

### 2.1 Description of the study area

This study covers Punjab and Sindh in Pakistan. These provinces are most populous. Sindh's population climbed 2.8 percent annually from 30.4 million in 1998 to 42.4 million in 2010. UNDP reports acute poverty and hunger in this province (Lian et al., 2023, Shahbaz et al., 2022a). Punjab has 60 % of the country’s population (Shahzad et al., 2021, Shahzad et al., 2019). Punjab’s industrial and agrarian base generates 58 % of Pakistan's GDP. These provinces have the best economies. Both provinces are susceptible to COVID-19. The local populace has been often exposed to the COVID-19 pandemic's adverse effects (Shahbaz et al., 2022b, Shahzad et al., 2022).

### 2.2 Data source and survey instrument

This study used an online poll to acquire primary data. The survey was translated into local Urdu language to help respondents understand it. The survey questionnaire was then incorporated into Google sheets^1^ using Facebook and WhatsApp and shared on social media to collect data. The study requires aged 17-years and older participants. The questionnaire claimed to use participants' data for education and research. Data confidentiality was also assured.

### 2.3 Selection of respondents

To eliminate sampling bias, this study used Cochran's (Causeur, 2005) approach for large populations to determine an ideal sample size. This equation represents the Cochran formula:

$$N=\;Z^{2}pq/e^{2}$$

Where N is the number of population samples, Z is the abscissa of the normal curve that cuts off portion of the tail of curve, e is precision, and p is predicted proportion of an attribute. The confidence interval is p = 0.5 (highest variability), precision level is 3 %, and Z attribute is 1.96.

$$N=\;{\left(1.96\right)}^{2}0.5\left(0.5\right)/{\left(0.03\right)}^{2}$$

N=0.9604/0.0009

N≈1067

A sample size of 1067 was estimated for this study to be the appropriate size for the survey.

### 2.4 Survey design

Following Haq et al. (Cheng et al., 2022, Haq et al., 2020a, Haq et al., 2020b, Lian et al., 2022), we utilized a non-probability snowball sampling method to collect data. The survey in the 4th pandemic wave from Punjab and Sindh provinces collected 1067 responses from July to October 2021. COVID-19 prevention methods like “stay at home and practice social distancing” made data collection from responders the hardest task. This non-probability-based method collects samples in random sequence and has some advantages. Investigators save time and money using this method. This method was used because respondents were inaccessible (Haq et al., 2020a, Haq et al., 2020b), meeting people one-on-one was difficult during disease (Etikan, 2016).

### 2.5 Analytical approaches

#### 2.5.1 Model for poverty estimates

According to the purchasing power parity 2011, an international poverty line of $1.90 per day is considered poverty. In order to measure the per capita income of a person, the following formula was used.

$$\mathrm{Per}\;Capita\;Income=\;\frac{\mathrm{Total}\;Gross\;Income}{\mathrm{Family}\;Size}$$

Moreover, in order to estimate the peoples per capita food expenditures, the mathematical formula used was following.

$$\mathrm{Per}\;Capita\;Food\;Expenditures=\;\frac{\mathrm{Total}\;Expenditures\;on\;Healthy\;Diets}{\mathrm{Family}\;Size}$$

The World Bank study (Dizon et al., 2019) estimated healthy eating costs. The cost of a healthy diet was calculated using FAO dietary criteria. In Pakistan, necessary healthy cost PKR 87.00 per day (2019 US$). Thus, this study analyzed and subtracted the current per capita food expenditures on healthy diets per day from the threshold food expenditure PKR 87.00 to estimate the number of people who cannot afford a healthy diet.

#### 2.5.2 Structural equation model for correlations

The mathematical form of the structural relationship between the independent variable, mediator, and dependent variables, following (Chandio et al., 2021, Cheong et al., 2003, Pazienza and De Lucia, 2020, Pratibha et al., 2019), is as follows:

η2 = Bη1 + Γξ + ζ.where ‘η2′ for impacts (endogenous), ‘η1′ for food purchasing cost, COVID-19, health, and food consumption factors (mediator variables), and ‘ξ’ for socioeconomic characteristics (exogenous). ‘B’ denotes the direct impact of food purchasing cost and consumption on health, while ‘Γ’ reflects correlations between food purchasing cost and health parameters. ‘ζ’ represents model measurement error. Table 2 describes model variables.

**Table 2:** The variable construct and description of variables included in the questionnaire used in COVID-19 pandemic online survey for this study’s data in Pakistan from July to October 2021.

	Description	Measurement	Variable	Construct
1	COVID-19 causes job losses, do you think?	Perceived probability: 1–10 scales	Employment loss	Food Cost Variables
2	COVID-19 causes loss of working hours?	Numerical digital	Loss of work hours	
3	Do you think COVID-19 causes food inflation and increases food prices?	Perceived probability: 1–10 scales	Food inflation	
4	Do you think COVID-19 increase your debt rate?	Perceived probability: 1–10 scales	Debt increase	
5	Do you think COVID-19 have an impact on your business?	Perceived probability: 1–10 scales	Business impact	
6	Have you obtained financial aid?	Yes, No	Financial aid	
7	Do you have a refrigerator for food storage?	Yes, No	having refrigerator	Food Consumption
8	Do you have access to basic necessities such as clean water and sanitation?	Yes, No	basic necessities	
9	Did you panic during the COVID-19 pandemic?	1–5 scales for each assessment (Extremely – Not at all	Panic	
10	Were you worried during the COVID-19 pandemic?	1–5 scales for each assessment (Extremely – Not at all	Worried	Health factors
11	Were you depressed during the COVID-19 pandemic?	1–5 scales for each assessment (Extremely – Not at all	Depressed	
12	My family members were under stress?	1–5 Likert scales (Always – Never)	family Anxiety	
13	do you stressed due to the loss of existing work and struggle to find a new job during the pandemic?	1–5 Likert scales (Always – Never)	Work stress	
14	What is the location of the area with regard to covid-19 cases?	More than 1000 cases, OR less than 1000 cases	Geographical area	
15	Were you in lockdown during the pandemic?	Yes, No	Lockdown	COVID-19 factors
16	Do you think you may contain the virus?	Perceived probability: 1–10 scales	perceive infection	

## 3 Results of the study

### 3.1 Descriptive statistics

According to the results, 40.9 % of respondents are from rural areas and 59.1 % from urban areas. The study accurately represents women with 40.2 % female and 59.7 % male respondents. On average, 55 % of responders come from 5 to 8-person families. For 68.9 % and 24.2 % of respondents have one or two earning family members. About 78.9 % of households own a refrigerator. And, 81.8 % couldn't afford the food, and 9.2 % believed it was costly. Only 27.6 % of respondents have formal work, yet 49.8 % are laborers (Table 3).

**Table 3:** Distribution of socioeconomic features of respondents aged 17 and older who completed online survey for COVID-19 effect evaluations for this study in Pakistan from July to October 2021.

Variable	Description	N	%
Location	Rural	436	40.9
	Urban	631	59.1
Gender	Female	429	40.2
	Male	638	59.7
Joint family		844	79.1
Nuclear/separate family		223	20.9
Family size	1–4	93	8.7
	5–8	587	55
	8–12 and above	387	36.3
Earning members	1	735	68.9
	2	258	24.2
	3	62	5.8
	4 and above	12	1.1
Having refrigerator	No	225	21.1
	Yes	842	78.9
Is food affordable?	Expensive	873	81.8
	No	95	8.9
	Yes	99	9.2
	Formal employment	294	27.6
Type of Employment	Informal employment	242	22.6
	Labor	531	49.8

### 3.2 Food consumption, poverty incidence, and cost of purchasing food in era of COVID-19

a) Food consumption

**Fig. 2:**
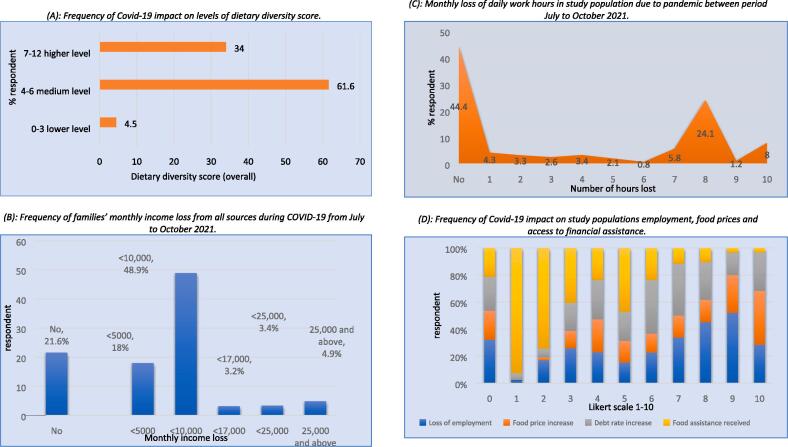
Frequency of the study population exposure with the effects of COVID-19 in Pakistan from July to October 2021.

Food consumption was measured using dietary diversity score of a family (Fig. 2A). Around 4.5 % and 61.6 % of respondents, respectively, have lower and medium dietary ratings. While only 34 % of individuals grade their dietary diversity higher.

b) Income

The income loss corresponds to Pakistani rupees (pkr) (177 pkr = $1 in 2021). Up to 10,000 Pakistani rupees were lost by 48.9 % of respondents. The COVID-19 epidemic in the research area costs 18 % of people 5,000 pkr per month in direct income. However, only 21.6 % reported no direct income loss. While 3.4 % and 4.9 % reported income losses of 25,000 pkr or more respectively (Fig. 2B).

c) Work hours

Nearly 55.6 % of respondents indicated a loss of work hours of 1 to 10 h per day (workday loss is measured in hours/day), with 24.1 % reporting a maximum of 8 h per day. 44.4 % did not report any loss. Thus, the COVID-19 pandemic in Pakistan reduced working hours and income (Fig. 2C).

d) Employment, food prices, and financial assistance

Fig. 2D shows COVID-19′s perceived effects on a 1-to-10 scale. On scale of “10,” 64.9 %, 46.2 %, and 46.3 % of people reported food price increase, loss of employment, and debt rate increase had the greatest impact on their lives during the disease which indicates that impact of poverty on food purchasing causes food and nutrition insecurity. With 64.9 % of respondents reporting a 10th scale increase in food prices, just 4.9 % reported receiving food assistance as mitigation.

e) Incidence of poverty

This study used the global poverty line of $1.9 to determine the number of people living below it. Table 4 shows that 9.38 % of people spend less than $1.9 daily (1$=177 pkr in 2021). Spending under $1.9 increased from 9.38 to 50.42 percent. This indicates that COVID-19 increased poverty in the studied area by 41.04 %.

**Table 4:** Measurements of the incidence of poverty during COVID-19 disease period in Pakistan: July-October 2021.

Affordability measures	PreCovid-19	During Covid-19	Change
Below PKR 366 ($1.9)	100	538	+438
	9.38	50.42	+41.04
Above PKR 366 ($1.9)	967	529	−438
	90.62	49.58	−41.04

f) Minimum cost on healthy diets

Findings showed 23.15 % people cannot afford healthy food everyday. Compared to 16.5 % of non-infected people, 6.56 % of infected people cannot afford the daily minimum cost of healthy meals (pkr 87 as determined by World Bank).

Table 5 shows about 5.5 %, 10.7 %, and 6.7 % of formal, informal, and labour class workers cannot afford minimum daily food expense. The largest daily per capita expenses are for informal labourers. Almost 46.6 percent of men and 33.6 % of women in the study area cannot afford the minimum daily food expense, indicating high poverty. Outdoor workers ate less healthy food than isolated people. And, for basic sustenance, 19.8 % of working people (Table 6) couldn't afford pkr 87.

**Table 5:** Measurements of the frequencies of minimum cost on healthy diets with respect to sociodemographic characteristics among study participants in Pakistan during the COVID-19 pandemic: July-October 2021.

Measurement Variable	Per capita expenditure on healthy food (Below threshold PKR 87)		Per capita expenditure on healthy food (Above PKR 87)	
	N	%	N	%
Per capita expenditure on healthy food	247	23.15	820	76.85
Infected	70	6.56	420	39.36
Non-infected	176	16.5	401	37.58
Fomral employed labor	59	5.5	236	22.1
Informal employed labor	114	10.7	128	12
Gross Labor	72	6.7	458	43
Female	359	33.6	70	6.6
Male	497	46.6	141	13.2
Working	212	19.8	755	70.8
Isolation	33	3.1	67	6.3

**Table 6:** Estimation of parameters and covariances between the variables of food purchasing cost, food consumption, COVID-19, and health factors in Pakistan in 2021.

Endogenous variables	Exogenous variables	Standardized path coefficient	SE	Z	P>│z│
Food purchasing cost factors	Health effect	0.5208***	0.0313	16.62	0.000
	COVID-19	−3.921***	0.5438	−7.21	0.000
Health factors	Food consumption	0.0523***	0.0159	3.29	0.001
Food consumption factors	Food purchasing cost	0.0551	0.0820	0.67	0.501
COV (food purchasing cost, food consumption)		-0.2451	0.0410	−5.98	0.000
COV (food purchasing cost, COVID-19)		0.8959	0.1346	6.65	0.000
COV (Health factors, COVID-19)		0.0974	0.0142	6.84	0.000

Note: *** significant at, 1% level.

### 3.3 Structural relationships

The results revealed that SEM (Table 7) data show an excellent model fit, (χ2 = 1693.61 (Prob > Chi2 = 0.000 < 0.001), α = 5 %, loglikelihood = -24574). The regression coefficient COVID-19 factors on food cost is negative and statistically significant, while food cost on health is positive and significant, indicating that the COVID-19 pandemic reduced people's ability to buy food. The coefficient of health factors on food consumption is positive and statistically significant, indicating that better food consumption improves health and vice versa. Finally, the regression coefficient of food consumption cost on food purchasing cost is positive and statistically significant, indicating that higher-income persons can afford and consume more food.

**Table 7:** The findings of regression analysis using structural equation model for the correlations between COVID-19 effects on health and food purchase cost variables in Pakistan in 2021.

Endogenous variables	Exogenous variables	Coefficient	SE	Z	P > │z│
Food purchase cost Variables	Employment loss	0.0165	0.0090	1.84	0.066
	Loss of work hours	-0.0110	0.0062	−1.79	0.074
	Food inflation	0.0221	0.0106	2.08	0.037
	Debt increase	0.0037	0.0106	0.36	0.722
	Business impact	0.0167	0.0255	0.66	0.511
	Financial aid	-0.0442	0.0433	−1.02	0.308
	Constant	3.705	0.2675	13.85	0.000
Health factors	Panic	0.0243	0.0251	0.97	0.333
	Worried	0.0748	0.0346	2.16	0.031
	Depressed	0.2811***	0.0306	9.18	0.000
	Family anxiety	0.5138***	0.0367	13.99	0.000
	Work stress	0.3506***	0.0303	11.54	0.000
	Constant	−1.542	0.1410	−10.94	0.000
Food consumption factors	Having refrigerator	0.4150***	0.1305	3.18	0.001
	Basic necessities	0.1997***	0.2356	0.85	0.000
	Constant	5.447	0.2799	19.46	0.000
COVID Factors	Geographical area	0.0064	0.0075	0.85	0.394
	Lockdown status	-0.0076	0.0140	−0.54	0.587
	Perceived infection	-0.0059***	0.0022	−2.67	0.008
	Constant	0.4784	0.0240	19.90	0.000
Var (e.income effect)		3.6922	0.9907		
Var (e.health effect)		0.6259	0.0302		
Var (e. DDS)		2.7977	0.1213		
Var (e.COVID)		0.24753	0.0106		
Prob > Chi2 = 0.000		LR test Chi2 = 1693.61		Log likelihood = -24574.752	

Note: *** significant at, 1% level.

### 3.4 Regression correlates

The employment loss, debt growth, business impact, and health effects are positive and statistically significant, indicating that the food purchasing cost and financial trouble of the people are significantly related to their economic circumstances. If financial conditions worsen during the COVID-19 pandemic, panic, depression, worries, and other stresses increase, causing health problems. For instance, higher stress levels during COVID-19 are positively linked and statistically significant. Food consumption is positively and significantly correlated with the possession of a refrigerator for food storage, food purchasing cost under strict lockdown, and access to basic supplies like clean drinking water and better sanitation. This shows that higher-income people with more purchasing power can afford and consume more food.

## 4 Discussion

In COVID-19, people lost substantial amount of income, work hours, and the expense on healthy diets, as well as food purchasing ability. Informal laborers, members of the working class, women, the elderly, and those under stress are more likely to be unable to afford a healthy diet. This study's findings are consistent with those of (Hall et al., 2021, Hall, 2021, Jatav et al., 2022). People adopted mitigating measures regarding food consumption. The lack of food and income affects impoverished residents' health and raises COVID-19 infection rates. Policies should focus on ensuring immediate and sustainable food supplies to counteract the effects of rising food costs. People are becoming poorer and more food insecure, (Zidouemba et al., 2020) stressed emergency relief. Marginalized population minimized income losses by obtaining loans and cutting savings and non-food expenditures (Headey et al., 2020). Thus, each nation must prepare food business for global disasters, and public health programs should focus on direct food subsidies for low-income households. COVID-19-related food insecurity worried international humanitarian organizations even beyond disease period (Hirvonen et al., 2021). Due to growing food prices, hunger and malnutrition increased, which might have dire effects for millions of people in the world's most vulnerable countries whose families cannot afford basic meals. The aftermath of the COVID-19 epidemic was worse than the virus itself. Due to COVID-19′s job losses and lower income, millions of families are forced to miss meals, buy cheaper, less nutrient-dense food, or stop eating entirely, which is unhealthy (Shahzad et al., 2021). The economic downturn and poverty increase make the COVID-19 pandemic's effects on the Sustainable Development Goals (SDGs-2) and food insecurity last longer, limiting food availability and accessibility even after the pandemic (Udmale et al., 2020).

## 5 Conclusion

COVID-19 can be mitigated by giving food assistance. COVID-19 caused poverty in the study area, and around 23 % of the people couldn't afford healthy food everyday. Women, informal employees, the working class, and non-infected people had more trouble affording healthy meals. Health problems plagued food insecure people. The SEM model shows that food purchasing cost is positively correlated with health, COVID-19 variables are negatively associated with it. Health considerations affect food consumption positively and significantly. Finally, food purchasing cost and consumption are positively and statistically significantly connected. It suggests that higher-income people can buy food, eat more, and have fewer health issues. During COVID-19, food aid increased to offset negative food cost shocks. Our findings showed that COVID-19 income shocks decreased people's incomes, making poverty more widespread.

Policy Implications

This study stressed that better food assistance programs were needed, and that the community had a responsibility to remove barriers to food access during public health emergencies, including physical and financial accessibility. Our research showed that many people lost income-earning opportunities. Thus, the government should provide short-term financial transfers or subsidies. Economic expansion is a better long-term plan since it increases food purchasing ability and gives the poor access to jobs, food, and healthcare. The afflicted community needs to implement Pakistan's Ehsas Income Program, which provides monetary help to low-income people.

Funding Statement

This research is supported by the Postdoctoral Research Funds of School of Economics and Trade, Hunan University in 2023.

Ethical Considerations

This research doesn’t harm any human or animal species and has no harmful impact on the environment.

## CRediT authorship contribution statement

**Muhammad Aamir Shahzad:** Conceptualization, Methodology, Software, Resources. **Lianfen Wang:** Supervision, Writing – review & editing. **Shengze Qin:** Data curation, Writing – original draft, Software, Validation. **Sha Zhou:** Visualization, Investigation.

## Declaration of Competing Interest

The authors declare that they have no known competing financial interests or personal relationships that could have appeared to influence the work reported in this paper.

## Data Availability

The data that has been used is confidential.

## Footnotes

1 The data was collected by using the following google sheets survey web links:Survey 1 Link: https://docs.google.com/forms/d/e/1FAIpQLSeLIk2E-ayrRB2FpKSjdLezCv3zyfKyPlIEqHVlCK3irxqHjQ/viewform?usp=sf_linkSurvey 2 Link: https://docs.google.com/forms/d/e/1FAIpQLSdJ6edTgJkPYuwKTu8-1VXZRtIzU3MW2LbVML9HJKK1jNVZwA/viewform?usp=sf_link.
